# Live birth prevalence of hemolytic disease of the fetus and newborn in the United States from 1996 to 2010

**DOI:** 10.1016/j.xagr.2023.100203

**Published:** 2023-03-24

**Authors:** Devin Yu, Leona E. Ling, Alexis A. Krumme, May Lee Tjoa, Kenneth J. Moise

**Affiliations:** 1 Janssen Research & Development, LLC, Cambridge, MA (Drs Yu, Ling, Krumme, and Tjoa); 2 The Department of Women's Health, Dell Medical School, The University of Texas at Austin, Austin, TX (Dr Moise).

**Keywords:** alloimmunization, blood disorder, cesarean delivery, feto-maternal hemorrhage, hemolytic disease of the fetus and newborn (HDFN), hospital discharge status, hospital length of stay, inpatient visits, National Hospital Discharge Survey, observational cohort study, retrospective

## Abstract

**BACKGROUND:**

Hemolytic disease of the fetus and newborn (HDFN) is mediated by maternal alloantibodies, a consequence of immune sensitization during pregnancy with maternal-fetal incompatibility with ABO, Rhesus factor (Rh), and/or other red blood cell antigens. RhD, Kell, and other non-ABO alloantibodies are the primary cause of moderate to severe HDFN, whereas ABO HDFN is typically mild. HDFN live birth prevalence owing to Rh alloimmunization among newborns in the United States was last estimated to be 106 per 100,000 births in 1986. HDFN live birth prevalence owing to all alloantibodies was estimated to be 817 to 840 per 100,000 in Europe. There is a need for updated prevalence estimates in the United States and a better understanding of disease demographics, severity, and treatments.

**OBJECTIVE:**

This study aimed to estimate the live birth prevalence of HDFN and the proportion of severe cases of HDFN in the United States, to describe the associated risk factors, and to compare the clinical outcomes and treatments among healthy newborns, newborns with HDFN, and newborns who are sick without HDFN using a nationally representative hospital discharge database.

**STUDY DESIGN:**

In this retrospective, observational cohort study, we used data from the 1996 to 2010 National Hospital Discharge Survey to identify live births, defined by inpatient visits with the newborn flag, with and without a diagnosis of HDFN across 200 to 500 sampled hospitals (≥6 beds) per year. Patient and hospital characteristics, alloimmunization status, disease severity, treatment, and clinical outcomes were evaluated. Frequencies and weighted percentages were calculated for all variables. Logistic regression was used to compare the characteristics between newborns with HDFN and other newborns using odds ratios.

**RESULTS:**

Of 480,245 live births identified, 9810 HDFN cases were recorded. When weighted to the United States population, this corresponded to a live birth prevalence of 1695 per 100,000 live births. Compared with other newborns, newborns with HDFN were more likely to be female, Black, living in the South (vs the Midwest or West), and treated at larger (>100 beds) and government-owned hospitals. ABO and Rh alloimmunization accounted for 78.1% and 4.3% of newborns with HDFN, respectively, whereas HDFN caused by other antigens, such as Kell and Duffy, accounted for 17.6% of the cases. Among newborns with HDFN, 22% received phototherapy, 1% received simple transfusions, and 0.5% received exchange transfusions or intravenous immunoglobulin. Newborns affected by HDFN caused by Rh alloimmunization were more likely to require medical interventions, including simple or exchange transfusions, and more likely to be delivered by cesarean delivery. Overall, HDFN was associated with a longer hospital length of stay in the neonatal intensive care unit when compared with healthy and other sick newborns, a higher rate of cesarean delivery, and a higher rate of nonroutine discharge than healthy newborns.

**CONCLUSION:**

Overall, the live birth prevalence of HDFN was higher than those previously reported, whereas Rh-induced HDFN live birth prevalence was similar to those previously reported. HDFN live birth prevalence owing to Rh alloimmunization decreased over time, likely because of continued Rh immune globulin prophylaxis. Treatment patterns for newborns with HDFN and the comparative clinical outcomes when compared with healthy newborns confirm the continued clinical needs of this population.

**AJOG Global Reports at a Glance**

**Why was this study conducted?**

This study aimed to determine the live birth prevalence of Rhesus factor (Rh)-induced hemolytic disease of the fetus and newborn (HDFN) in the United States, which was last estimated to be 106 per 100,000 births in 1986.

**Key findings**

The live birth prevalence of HDFN was 1695 per 100,000 births; Rh-induced HDFN live birth prevalence rates ranged from 44.4 to 98.7 per 100,000 births. Patient-related risk factors were female gender, Black race, and living in the South. Hospital-related risk factors were size (>100 beds) and government ownership. Newborns with HDFN were more likely to be delivered by cesarean delivery and have a hospital stay >2 days than healthy newborns.

**What does this add to what is known?**

This study provides the most recent estimate of the live birth prevalence of HDFN in the United States, which is consistent with the live birth prevalence rates previously reported in similar global demographic populations with a trend toward decreasing RhD alloimmunization after introduction of anti-D prophylaxis.

## Introduction

Hemolytic disease of the fetus and newborn (HDFN) occurs when maternal-fetal red blood cell antigen incompatibility induces maternal anti–red blood cell alloantibodies. These alloantibodies transfer across the placenta to the fetus and can persist for months in the newborn and infant, causing hemolysis, hyperbilirubinemia, and anemia.^1^ Alloimmunization with Rhesus factor (Rh) D, Rhc, or Kell antibodies can place the fetus at risk for severe anemia requiring intrauterine transfusions, hydrops fetalis, or fetal demise.^2^ In newborns, short- and long-term clinical consequences may include jaundice, kernicterus, anemia, thrombocytopenia, and—in rare cases—serious neurodevelopmental outcomes.^1^^,^^3^ Treatment of severe HDFN in newborns includes phototherapy for jaundice, simple red blood cell transfusion for anemia, and exchange transfusion or neonatal intravenous immunoglobulin (IVIG) for escalating jaundice with risk of kernicterus.^3^, ^4^, ^5^

There are no recent nationwide studies in which the live birth prevalence of HDFN caused by Rh, ABO, and other or unknown blood antigens in the United States were estimated. The last reported national rate of Rh-induced HDFN was 106 per 100,000 births in the 1980s.^6^ Although there have been more recent ex–US-based studies reporting live birth prevalence rates of all cases of HDFN among newborns and HDFN caused by specific antigens in newborns and maternal alloimmunization during pregnancy, these studies may not be generalizable to the US-based population because of varying sociodemographic factors and standards of medical care.^7^^,^^8^ Several single-center studies have estimated that the live birth prevalence of maternal alloimmunization ranged from 400 to 1200 per 100,000 births.^9^, ^10^, ^11^, ^12^ Variability in these estimates may be reflective of inconsistent inclusion criteria and small sample sizes; moreover, studies of alloimmunized pregnancies do not include cases of HDFN that were only recognized at birth.

Little is known about the characteristics of newborns with HDFN in the United States, including demographic features, disease severity, and treatment needs during the perinatal period. Although several single-center, US-based studies have provided estimates of maternal alloimmunization, the characteristics of newborns with HDFN may not be generalizable to populations outside of tertiary medical centers.^10^^,^^12^^,^^13^

The 3 study objectives were to estimate the live birth prevalence of all HDFN, Rh factor alloimmunized HDFN only, and clinically severe HDFN; to identify clinically significant risk factors for HDFN; and to determine the rates of clinical outcomes and treatments associated with newborns with HDFN when compared with healthy newborns and other sick newborns. Using data from the 1996 to 2010 National Hospital Discharge Survey (NHDS), this study aimed to provide a nationally representative estimate and characterization of newborn HDFN in the United States.

## Materials and Methods

### Study design and data source

This retrospective, observational cohort study used data collected as part of the 1996 to 2010 NHDS. This annually conducted national probability survey is a representative sample of US-based discharges from 200 to 500 noninstitutional, short-stay general hospitals with ≥6 beds across 50 states.^14^ Survey sampling was completed in a 3-stage probability design that accounted for geographic location, hospital size, and number of discharges. Data were transcribed or electronically gathered from medical records.

The data sets were entered, edited, and weighted annually by the National Center for Health Statistics (NCHS) and contract companies. Available variables include maternal age and neonatal race, sex, geographic region, discharge status, hospital size (number of beds), hospital ownership, and the *International Classification of Diseases, Ninth Revision, Clinical Modification* (ICD-9-CM) diagnosis and procedure codes. The NHDS has been used in analyses related to childbirths and newborns, including newborn circumcisions,^15^ childbirth hospital stays,^16^ and preeclampsia.^17^

### Study population

Births, also referred to as newborn cases, were identified using the newborn flag and were verified using an age of 0 days. Newborns with HDFN were identified by ≥1 ICD-9-CM diagnostic code for HDFN (ie, 773.0–773.5). Newborns with suspected coding errors (eg, multiple and different) were excluded. This study did not capture fetal cases of HDFN that ended in fetal demise. Other sick newborns were defined as any newborn with an ICD-9-CM diagnosis code that was not 773.0 through 773.5; healthy newborns were defined as any newborn with only a birth-related and no other ICD-9-CM diagnostic code.

### Outcomes

#### Live birth prevalence of HDFN and HDFN severity

HDFN and severe HDFN live birth prevalence rates in a US-based population were determined using the weighted frequency of newborns with the relevant ICD-9-CM codes divided by the weighted frequency of all newborns as provided by the NCHS.^18^ Severe cases were defined as HDFN newborns with specific codes for hydrops fetalis, kernicterus, and/or late anemia (773.3–773.5) and/or cases requiring exchange transfusion or IVIG (procedure codes: 99.01 and 99.14) during the newborn stay.

#### Patient and visit characteristics

Newborn characteristics of interest were sex, race, and geographic region. Hospital characteristics of interest were hospital size (ie, number of beds), hospital ownership, and expected source of payment.

#### Alloimmunization type

Alloimmunization type was identified using the ICD-9-CM diagnosis codes for Rh alloimmunization (773.0), ABO alloimmunization (773.1), and other or unspecified alloimmunization (773.2). For cases with multiple codes, any combination with Rh alloimmunization was grouped as Rh alloimmunization. Any combination with ABO, except when combined with Rh, was grouped as ABO. Visits without Rh or ABO codes, including singular codes for hydrops fetalis, kernicterus, and late anemia, were classified as other/unknown (Supplemental Figure 1).

#### Treatments

HDFN-related treatments were identified using the ICD-9-CM procedure codes and included phototherapy (ultraviolet light therapy [99.82], other phototherapy [99.83]), simple transfusion (99.04), exchange transfusion (99.01), and IVIG (99.14).

#### Clinical outcomes

Clinical outcomes of interest included birth method (cesarean delivery or vaginal birth), determined using ICD-9-CM diagnosis codes (Supplemental Table 1), discharge status recorded as not routine, and hospital length of stay >2 days. ICD-9-CM codes also were used to identify newborns with HDFN (773.0–773.5), healthy newborns (no code), and newborns with other illnesses (001–999, except 773.0–773.5).

### Statistical analyses

The NCHS standards for reliability suggest that estimates should be based on ≥30 discharge records and have a relative standard error ≤30%. A total number of >9000 records of newborns with HDFN was identified, and the number of newborns with Rh-HDFN was >50. Comparisons for variables with <30 records are presented in the Supplemental Material.

For patient characteristics and disease severity, counts and proportions of newborns with HDFN were reported for each characteristic. Odds ratios, 95% confidence intervals (CIs), and *P* values were calculated as a comparison between the reference group and other groups for each characteristic using logistic regression.

For inpatient hospital visits, counts and weighted percentages with 95% CIs were calculated for newborns with and without HDFN and were compared using logistic regression. Weighted percentages were calculated using weighted frequencies from the NCHS. Weighted estimates were used because they allow extrapolation to national or regional estimates per the NHDS Data File Documentation. Visit characteristics were adjusted for all other variables.

For HDFN-related treatment and alloimmunizations, counts and weighted percentages were calculated, and comparisons between groups were conducted using logistic regression.

Clinical outcomes were compared between newborns with HDFN, healthy newborns, and other sick newborns using logistic regression. All statistical analyses were preformed using SAS (version 9.4; SAS Institute, Cary, NC).

## Results

### Prevalence of HDFN and HDFN severity

**Figure 1:**
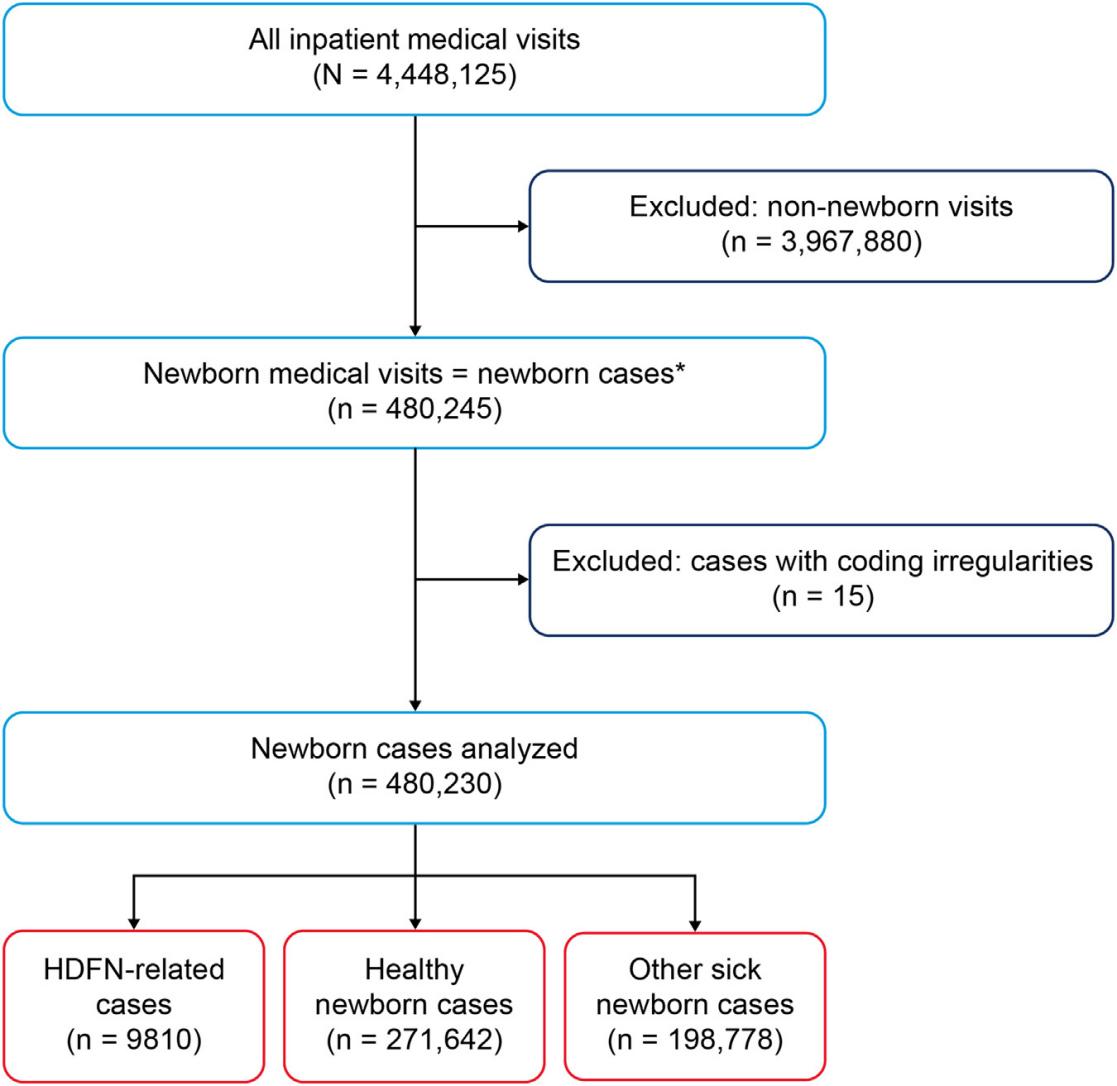
Schema for births and selection of study population *Asterisk* denotes the explanation for equivalence of visits and cases is provided in the section on study population in the Materials and Methods. *HDFN*, hemolytic disease of the fetus and newborn.

Between 1996 and 2010, 4,448,125 inpatient medical visits were recorded in the survey. After excluding non-newborn visits, 480,245 births were documented, representing 58,341,539 live births, with each of those considered to be an individual newborn case. Fifteen births were excluded owing to coding irregularities (Figure 1). A total of 9810 cases of HDFN, representing 988,568 cases nationally, were recorded, giving an HDFN live birth prevalence of 1695 cases per 100,000 births. Among newborns with HDFN, 0.6% (95% CI, 0.3–0.9) of cases were severe.

Yu. Live birth prevalence of hemolytic disease of the fetus and newborn in the United States from 1996 to 2010. Am J Obstet Gynecol Glob Rep 2023.

### Patient and visit characteristics

**Figure 2:**
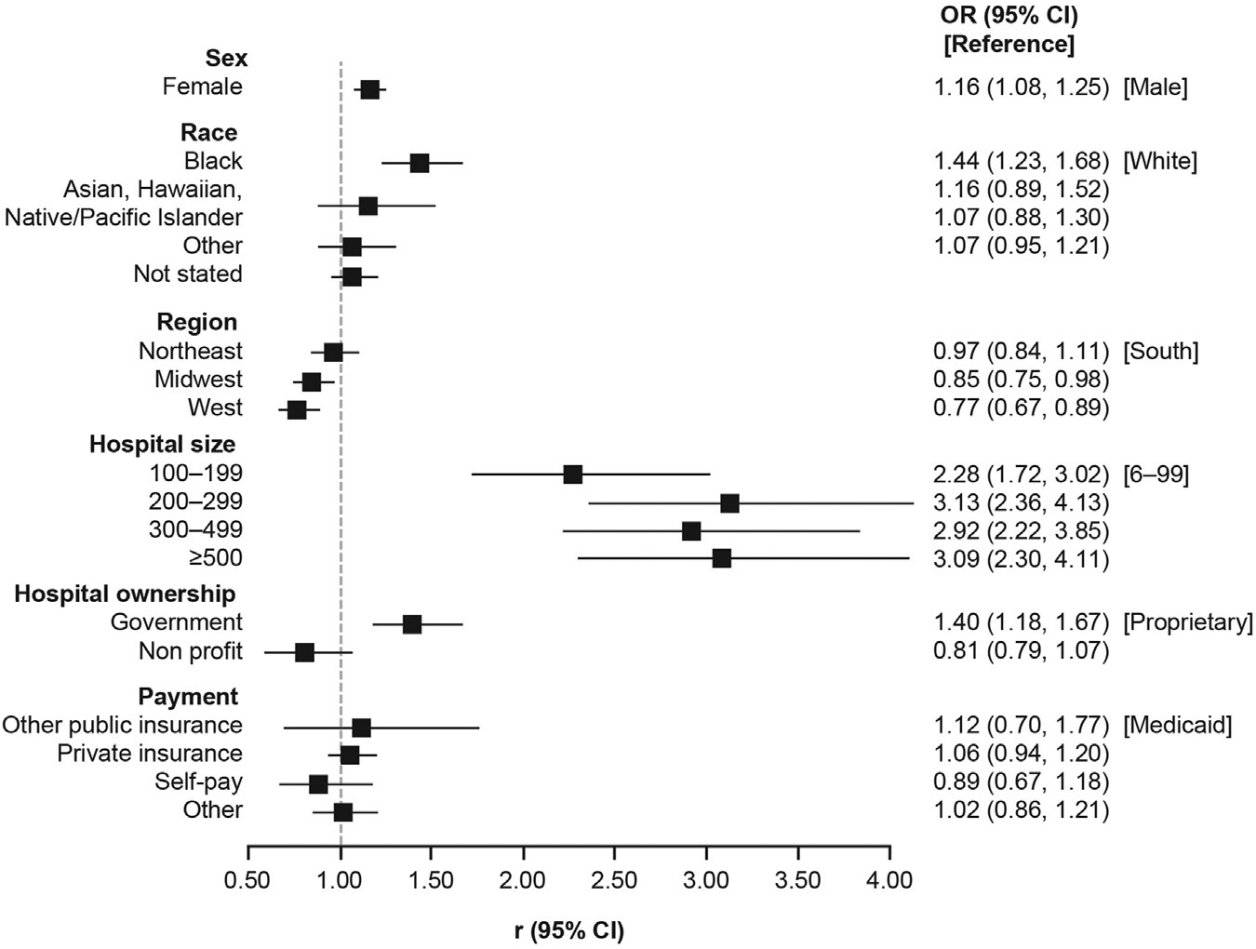
Risk factors associated with newborn HDFN diagnosis *CI*, confidence interval; *HDFN*, hemolytic disease of the fetus and newborn; *OR*, odds ratio.

Among newborns with HDFN, the majority were White, from the South, and discharged home (Table 1). Statistically significant patient-related risk factors for HDFN included female sex, Black race, and living in the South (vs in the Midwest or West). Statistically significant hospital-related risk factors associated with HDFN were size (>100 beds) and government ownership (Figure 2; Supplemental Table 2).

**Table 1:** Overview of HDFN newborn characteristics

Characteristic	Number^a^	Percentage (95% CI)^b^
Sex		
Male	4632	47.7 (45.9–49.5)
Female	5178	52.3 (50.5–54.0)
Race		
White	4339	50.1 (48.3–51.9)
Black	1647	18.2 (16.7–19.7)
Asian, Hawaiian, or Pacific Islander	246	3.5 (2.9–4.1)
Other	727	5.0 (4.3–5.6)
Not stated	2851	23.2 (21.9–24.6)
Region		
Northeast	2063	18.8 (17.4–20.2)
Midwest	2453	17.7 (16.6–18.9)
South	3837	43.0 (41.2–44.8)
West	1457	20.5 (19.0–22.0)
Birth method		
Vaginal delivery	6760	69.4 (67.7–71.0)
Cesarean delivery	3050	30.6 (29.0–32.3)
Discharge status		
Discharged home	9291	94.2 (93.3–95.1)
Not discharged home	140	1.9 (1.3–2.5)
Alive, disposition not stated	294	2.1 (1.7–2.5)
Dead	26	0.2 (0.1–0.3)
Not stated or not reported	59	1.6 (1.0–2.2)
Hospital size (number of beds)		
6–99	335	7.1 (5.9–8.4)
100–199	1818	20.8 (19.4–22.2)
200–299	2787	24.0 (22.5–25.6)
300–400	3292	31.5 (29.9–33.2)
≥500	1578	16.5 (15.2–17.7)
Hospital ownership		
Proprietary	912	10.4 (9.4–11.5)
Government	1134	18.2 (16.7–19.7)
Nonprofit	7764	71.4 (69.8–73.1)
Expected payment source		
Medicaid	3145	37.1 (35.3–38.9)
Other public insurance	111	1.7 (1.2–2.2)
Private insurance	4825	46.3 (44.5–48.1)
Self-pay	360	3.6 (3.0–4.3)
Other	1202	9.0 (8.1–9.9)

*CI*, confidence interval; *HDFN*, hemolytic disease of the fetus and newborn; *NHDS*, National Hospital Discharge Survey.

a Observed frequency

b Weighted national estimate from the NHDS.

Yu. Live birth prevalence of hemolytic disease of the fetus and newborn in the United States from 1996 to 2010. Am J Obstet Gynecol Glob Rep 2023.

### Alloimmunizations during pregnancy

**Figure 3:**
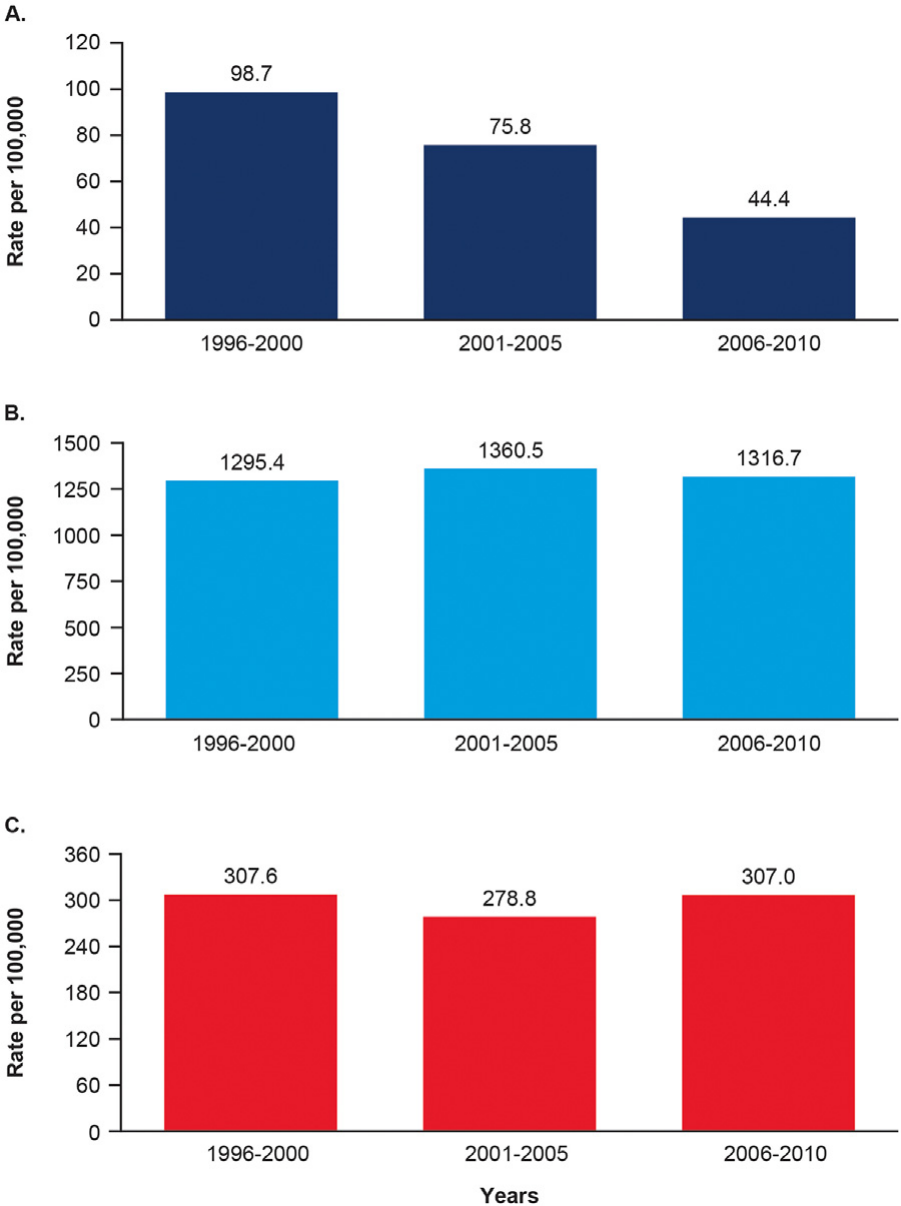
Rate of HDFN alloimmunization by year The Rh-HDFN alloimmunization rate (**A**), the ABO alloimmunization rate (**B**), and the alloimmunization rate for other or unknown antigen (**C**). *HDFN*, hemolytic disease of the fetus and newborn**.**

Among all newborns with HDFN, ABO alloimmunization was the most commonly reported type during pregnancy, accounting for 78.1% (95% CI, 76.7–79.6) of cases. Rh alloimmunization accounted for only 4.3% (95% CI, 3.6–4.9) of cases. The frequency of Rh-induced HDFN decreased over time, whereas that for ABO alloimmunization or other or unknown alloimmunizations remained consistent (Figure 3).

Yu. Live birth prevalence of hemolytic disease of the fetus and newborn in the United States from 1996 to 2010. Am J Obstet Gynecol Glob Rep 2023.

### Treatments

Among newborns with HDFN, 22% (95% CI, 20.5–23.5) received phototherapy, 1% (95% CI, 0.6–1.3) received simple transfusions, and 0.5% (95% CI, 0.2–0.8) received exchange transfusions and/or IVIG (Table 2). Newborns with HDFN with ABO alloimmunization during pregnancy were less likely to receive simple transfusions and exchange transfusions and/or IVIG than those with Rh alloimmunization during pregnancy (Supplemental Table 3).

**Table 2:** HDFN-related treatments

Treatment	Number^a^	Percentage (95% CI)^b^
Phototherapy		
Yes	2144	22.0 (20.5–23.5)
No	7666	78.0 (76.5–79.5)
Simple transfusion		
Yes	87	1.0 (0.6–1.3)
No	9723	99.0 (98.7–99.4)
Exchange transfusion and/or IVIG		
Yes	36	0.5 (0.2–0.8)
No	9774	99.5 (99.2–99.8)

*CI*, confidence interval; *HDFN*, hemolytic disease of the fetus and newborn; *IVIG*, intravenous immunoglobulin; *NHDS*, National Hospital Discharge Survey.

a Observed frequency

b Weighted national estimate from the NHDS.

### Clinical outcomes

**Figure 4:**
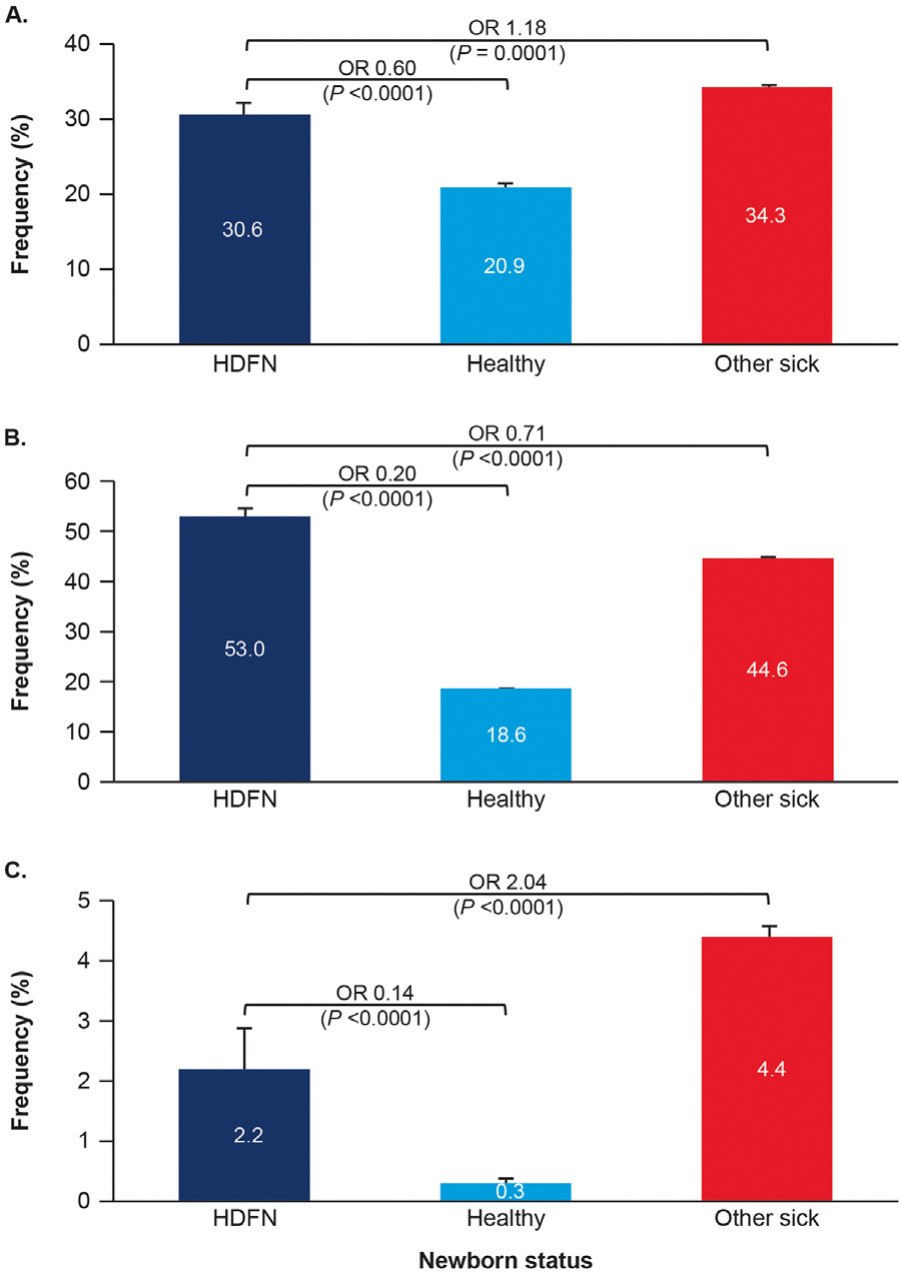
Frequency of clinical outcomes The outcome rates for cesarean delivery (**A**), hospital stay >2 days (**B**), and nonroutine discharge status (**C**) are presented. *HDFN*, hemolytic disease of the fetus and newborn; *OR*, odds ratio.

Compared with healthy newborns, newborns with HDFN were more likely to be delivered by cesarean delivery and to not be discharged routinely. However, when compared with other sick newborns, newborns with HDFN were less likely to be delivered by cesarean delivery and to not be discharged routinely. Newborns with HDFN were more likely to have a hospital stay >2 days than healthy newborns and other sick newborns (Figure 4).

Yu. Live birth prevalence of hemolytic disease of the fetus and newborn in the United States from 1996 to 2010. Am J Obstet Gynecol Glob Rep 2023.

## Comment

### Principal findings

Population-based analyses are needed to assess the overall burden of disease and to estimate gaps in the care of newborns with HDFN. Although some older and single-center studies have confirmed the persistent need for care of newborns with HDFN, recent nationwide HDFN live birth prevalence rates in the United States remain unconfirmed. The most recent nationwide estimate of Rh-induced HDFN dates back to the 1980s, shortly after the introduction of RhD immune globulin prophylaxis in the 1970s, which dramatically changed the rates of RhD alloimmunized (at-risk) pregnancies from approximately 13%–16% to 0.5%–1.8% after the introduction of postpartum administration of RhD immune globulin and to 0.14% to 0.2% with routine antepartum administration.^6^^,^^19^^,^^20^

The results of this study estimate that the live birth prevalence of all-cause HDFN is 1695 per 100,000 births, which is slightly higher than the recent Italian (817 per 100,000)^7^ and Bosnian (840 per 100,000) estimates.^8^ However, in the Italian study, rates were obtained through physician surveys with variable response rates; only 55% of physicians responded to the question regarding the rate of hemolytic disease of the newborn. In addition, data were provided by blood transfusion centers, which may represent only more clinically significant cases.^7^ Lower rates in the Bosnian study may be explained by the exclusion of newborns not admitted to the neonatal intensive care unit.^8^ These results may not be translatable to the United States because of differences in demographic and clinical characteristics between European and US-based patients.

Special attention may be given to Rh-induced HDFN, which is often more severe and requires more medical intervention.^21^ The last US-based national estimate of Rh-induced HDFN was 106 per 100,000 births based on hospital medical records of infants identified through the Centers for Disease Control and Prevention (CDC) Birth Defect Monitoring Program using ICD-9-CM codes and verified by evidence of a direct Coombs test, the antigen status of the mother and infant, and treatments listed in the medical records.^6^ Although the largest decline in Rh alloimmunization occurred in the 10 years following the approval of RhoGAM, the first anti-RhD prophylactic agent, in 1968,^22^ we observed a decline in the mean live birth prevalence of Rh-induced HDFN from 99 per 100,000 newborns in the period from 1996 to 2000 to 76 per 100,000 newborns from 2001 to 2005 and 44 per 100,000 newborns from 2006 to 2010. Although the reason for this decline is not clear and is beyond the scope of this study, additional guidelines for prevention of alloimmunization were instituted in the 1980s and 1990s, including recommendations for anti-D prophylaxis for alloimmunization events other than delivery, anti-D prophylaxis at 28 weeks’ gestation, and screening for feto-maternal hemorrhage.^23^^,^^24^ If correct, this explanation would support the idea that the Rh isoimmunization medical code is primarily being used for RhD alloimmunization. Lower recent rates are consistent with an Italian estimate of 55 per 100,000 deliveries in 2013 and an Icelandic estimate of 90 RhD alloimmunized pregnancies per 100,000 births.^7^^,^^25^ This supports previous research showing a decreasing but persistent live birth prevalence of RhD-alloimmunized HDFN, which continues to contribute to infant morbidity and mortality in the United States.^25^

Because of decreasing rates of RhD alloimmunization with anti-D prophylaxis, other blood antigens also are emerging as important causes of HDFN.^26^ We estimated that the live birth prevalence of HDFN caused by other or unknown antigens range from 278.8 to 307.6 per 100,000 births. This is consistent with the previously reported nationwide rate of non-RhD alloimmunization (Kell, Rh antibodies other than RhD, Duffy, Kidd, and Ss) of 328 per 100,000 in the Netherlands.^26^ In our analysis, other and unknown cases accounted for 17.6% of all captured HDFN cases. Similarly, a retrospective analysis of women treated with intrauterine transfusions for HDFN in Finland found that 17% were owing to non-RhD alloimmunizations (anti-c, -K, -E, -G, -C).^12^ Rates in a Greek study were notably higher at 710 per 100,000 pregnancies; however, rates of all HDFN also were reportedly higher in the Greek population, especially among migrant subpopulations.^27^ Overall, non-RhD alloimmunizations seem to account for significant proportions of newborns with HDFN.

Finally, our study found that newborns who were female, Black, and from the South had higher live birth prevalence rates of HDFN than newborns who were male, White, and from the West or Midwest. Notably, rates of diagnosis did not differ by insurance type. Racial differences in HDFN could be caused, in part, by the higher reported prevalence and severity of ABO incompatibility among Black individuals.^28^

### Results in the context of what is known

Guidelines by the American College of Obstetricians and Gynecologists recommend pre- and postnatal use of prophylactic RhD immune globulin in cases with a high risk for feto-maternal hemorrhage and other sources of alloimmunization.^29^ Despite the administration of RhD immune globulin, approximately 0.1% to 0.4% of women at risk become sensitized during pregnancy.^29^ This study confirms that a proportion of those alloimmunized pregnancies can be attributed to antigens other than RhD.

Our research confirms the persistence of HDFN and severe HDFN despite widespread use of RhD immune globulin prophylaxis. The severity is reflected by HDFN treatments, including neonatal phototherapy for jaundice, simple transfusions for neonatal anemia, and exchange transfusions of IVIG for risk of kernicterus.^3^, ^4^, ^5^ Top-off (simple) transfusions and exchange transfusions or IVIG treatment were approximately 10-fold and 4-fold lower, respectively, among newborns with ABO-induced HDFN than among newborns with Rh-induced or non-Rh–induced HDFN, consistent with the known risks in these groups.^5^^,^^7^ Our analysis showed that larger percentages of newborns with HDFN were delivered by cesarean delivery, had a nonroutine discharge, and had a longer hospital length of stay. Despite widespread availability of RhD prophylaxis and improved management, HDFN still significantly impacts neonates.

### Clinical implications

This study supports a low but persistent rate of RhD alloimmunization in the United States despite adoption of universal RhD prophylaxis and a steady rate of non-RhD alloimmunization with ABO and other non-RhD blood group incompatibilities. Increased rates of cesarean delivery, neonatal complications requiring longer hospital stays, nonroutine discharge, and, in rare cases, procedures indicative of severe neonatal disease suggest that there is a need for continued vigilance, training, and treatment options in the management of HDFN, particularly among patients of Black race and those in the Southern United States, who were observed to have higher live birth prevalence rates of HDFN in this study.

### Research implications

We described the demographic and clinical characteristics of newborns with HDFN in a US-based population. Further research is needed to explain the possible racial and geographic disparities among neonates that lead to different HDFN live birth prevalence rates among these cohorts. Moreover, future studies may explore clinical markers to predict who is at an increased risk for severe HDFN, which may assist physicians in the prevention and/or early identification of at-risk pregnancies. Finally, studies may be conducted to validate the definition of severe HDFN used in this study. Currently, definitions of HDFN severity may vary between studies; a unified definition will improve comparisons among future HDFN studies.

### Strengths and limitations

During the 15-year period of this survey, the NHDS captured >4 million inpatient visits, including 9810 HDFN-affected births, allowing for analysis of this rare disease. The NHDS database was created and organized by the CDC NCHS. The data were collected through a robust representative sampling method stratified based on geographic region and hospital size. Data were weighted by the NCHS to represent all US-based, in-hospital births.^18^^,^^30^ This large sample allowed us to analyze demographic characteristics, clinical outcomes, and trends over time. Our study has added to the knowledge base of HDFN, especially because only a few previous studies have thoroughly described patients with HDFN at a population level.

The NHDS has been used in the past to study pregnant^31^ and newborn^32^ populations. Furthermore, >98.8% of births in any given year from 1996 to 2010 occurred in hospitals.^33^ Thus, results from this study may be appropriately and reasonably generalized to most live births in the United States.

The accuracy of the ICD-9-CM coding could not be validated with detailed clinical information. Variability could occur in cases of unverified differential diagnoses based on presenting symptoms and unverified antigen testing.

The NHDS was designed to capture a representative sample of general hospital discharges—not to analyze rare fetal-maternal disease. Sampling is not stratified by primary, secondary, and tertiary centers, although severe HDFN may be more likely to be seen in the latter. In addition, previous research suggests a potential underestimation of neonatal birth defects when compared with the CDC Birth Defect Monitoring Program.^34^

Finally, the NHDS only captures live births and births in hospitals. HDFN, especially more serious cases, often manifests in the fetal stages. This study did not capture fetal-maternal care, cases ending in fetal loss, cases involving hydrops or stillbirth, or cases with newborns born outside of an inpatient hospital. The rate of fetal death caused by HDFN was calculated to be 0.39 per 100,000 live births based on data presented by Hoyert and Gregory.^35^ Thus, our study may underestimate the true burden of disease.

### Conclusions

The estimated rate and trend of HDFN reported here are consistent with those previously reported. The frequency of HDFN caused by Rh alloimmunization decreased over time, likely because of the widespread use of Rh immune globulin prophylaxis. Nevertheless, a low rate of Rh-induced HDFN persists, as does severe disease. Newborns with HDFN had a higher rate of cesarean delivery, a higher rate of nonroutine discharge, and a longer hospital stay than healthy newborns. Together, these results confirm the continued clinical needs of patients with HDFN. The demographic and clinical disparities identified merit further investigation.

## Appendix Supplementary materials

Image, application 1
