# Supplementary material for: Live birth prevalence of hemolytic disease of the fetus and newborn in the United States from 1996 to 2010
Source: AJOG Glob Rep. 2023 Mar 24;3(2):100203. doi: 10.1016/j.xagr.2023.100203 (PMC10205505; doi:10.1016/j.xagr.2023.100203)
Supplement: Supplementary file 1 [file mmc1.docx]

**Supplemental Material**

| **SUPPLEMENTAL TABLE 1. ICD-9-CM procedure codes for birth method** | |
| --- | --- |
| **Cesarean section birth** | |
| **Code** | **Description** |
| V30.01 | Single liveborn, born in hospital |
| V31.01 | Twin birth, mate liveborn, born in hospital |
| V32.01 | Twin birth, mate stillborn, born in hospital |
| V33.01 | Twin birth, unspecified whether mate liveborn or still born, born in hospital |
| V34.01 | Other multiple birth (≥3), mates all liveborn, born in hospital |
| V35.01 | Other multiple birth (≥3), mates all stillborn, born in hospital |
| V36.01 | Other multiple birth (≥3), mates liveborn and stillborn, born in hospital |
| V37.01 | Other multiple birth (≥3), unspecified whether mates liveborn or stillborn, born in hospital |
| V39.01 | Liveborn, unspecified whether single, twin or multiple, born in hospital |
|  | |
| **Vaginal birth** | |
| V30.00 | Single liveborn, born in hospital, delivered without mention of cesarean section |
| V30.1 | Single liveborn, born before admission to hospital |
| V30.2 | Single liveborn, born outside hospital and not hospitalized |
| V31.00 | Twin birth, mate liveborn, born in hospital, delivered without mention of cesarean section |
| V31.1 | Twin birth, mate liveborn, born before admission to hospital |
| V31.2 | Twin birth, mate liveborn, born outside hospital and not hospitalized |
| V32.00 | Twin birth, mate stillborn, born in hospital, delivered without mention of cesarean section |
| V32.1 | Twin birth, mate stillborn, born before admission to hospital |
| V32.2 | Twin birth, mate stillborn, born outside hospital and not hospitalized |
| V33.00 | Twin birth, mate stillborn, born in hospital, delivered without mention of cesarean section |
| V33.1 | Twin birth, mate stillborn, born before admission to hospital |
| V33.2 | Twin birth, mate stillborn, born outside hospital and not hospitalized |
| V34.00 | Other multiple births (≥3), mates all liveborn, born in hospital, delivered without mention of cesarean section |
| V34.1 | Other multiple births (≥3), mates all liveborn, born before admission to hospital |
| V34.2 | Other multiple births (≥3), mates all liveborn, born outside hospital and not hospitalized |
| V35.00 | Other multiple births (≥3), mates all stillborn, born in hospital, delivered without mention of cesarean section |
| V35.1 | Other multiple births (≥3), mates all stillborn, born before admission to hospital |
| V35.2 | Other multiple births (≥3), mates all stillborn, born outside hospital and not hospitalized |
| V36.00 | Other multiple births (≥3), mates liveborn and stillborn, born in hospital, delivered without mention of cesarean section |
| V36.1 | Other multiple births (≥3), mates liveborn and stillborn, born before admission to hospital |
| V36.2 | Other multiple births (≥3), mates liveborn and stillborn, born outside hospital and not hospitalized |
| V37.00 | Other multiple births (≥3), unspecified whether mates liveborn or stillborn, born in hospital, delivered without mention of cesarean section |
| V37.1 | Other multiple births (≥3), unspecified whether mates liveborn or stillborn, born before admission to hospital |
| V37.2 | Other multiple births (≥3), unspecified whether mates liveborn or stillborn, born outside hospital and not hospitalized |
| V39.00 | Liveborn, unspecified whether single, twin or multiple, born in hospital, delivered without mention of cesarean section |
| V39.1 | Liveborn, unspecified whether single, twin or multiple, born before admission to hospital |
| V39.2 | Liveborn, unspecified whether single, twin or multiple, born outside hospital and not hospitalized |

ICD-9-CM, *International Classification of Diseases, Ninth Revision, Clinical Modification*.

| **SUPPLEMENTAL TABLE 2. Hospital visit characteristics for HDFN-affected newborns** | | | |
| --- | --- | --- | --- |
| **Characteristic** | **Adjusted rate (per 100)^a^**  **(95% CI)** | **OR** | ***P* value** |
| Sex |  |  |  |
| Male (reference) | 1.6 (1.5-1.8) |  |  |
| Female | 1.9 (1.7-2.1) | 1.16 | <0.0001 |
| Race |  |  |  |
| White (reference) | 1.5 (1.4-1.7) |  |  |
| Black | 2.2 (1.9-2.5) | 1.44 | <0.0001 |
| Asian, Hawaiian, Native & Pacific Islander | 1.8 (1.4-2.2) | 1.16 |  |
| Other | 1.6 (1.4-1.9) | 1.07 |  |
| Not stated | 1.6 (1.5-1.8) | 1.07 |  |
| Region |  |  |  |
| South (reference) | 1.9 (1.7-2.1) |  |  |
| Northeast | 1.9 (1.7-2.1) | 0.97 |  |
| Midwest | 1.7 (1.5-1.9) | 0.85 | 0.01 |
| West | 1.5 (1.3-1.7) | 0.77 | <0.0001 |
| Hospital size, number of beds |  |  |  |
| 6-99 (reference) | 0.7 (0.6-0.9) |  |  |
| 100-199 | 1.7 (1.5-1.9) | 2.28 | <0.0001 |
| 200-299 | 2.3 (3.1-3.6) | 3.13 | <0.0001 |
| 300-400 | 2.2 (2.0-2.4) | 2.92 | <0.0001 |
| ≥500 | 2.3 (2.0-2.6) | 3.09 | <0.0001 |
| Hospital ownership |  |  |  |
| Proprietary (reference) | 1.6 (1.4-1.8) |  |  |
| Government | 2.2 (2.0-2.5) | 1.40 | <0.0001 |
| Nonprofit | 1.5 (1.3-1.6) | 0.81 |  |
| Expected source of payment |  |  |  |
| Medicaid (reference) | 1.7 (1.6-1.9) |  |  |
| Other public insurance | 1.9 (1.4-2.6) | 1.12 |  |
| Private insurance | 1.8 (1.7-2.0) | 1.06 |  |
| Self-pay | 1.5 (1.3-1.9) | 0.89 |  |
| Other | 1.8 (1.6-2.0) | 1.02 |  |
| Not stated | 1.6 (1.3-2.1) | 0.94 |  |

CI, confidence interval; HDFN, hemolytic disease of the fetus and newborn; OR, odds ratio.

^a^Rate of all-cause HDFN adjusted for other demographic/hospital variables.

| **SUPPLEMENTAL TABLE 3. Treatments by alloimmunization type** | | | |
| --- | --- | --- | --- |
| **Treatment** | **Adjusted rate (per 100)  (95% CI)** | **OR** | ***P* value** |
| Phototherapy |  |  |  |
| Rh (reference) | 21.9 (16.1-29.1) |  |  |
| ABO | 23.0 (21.3-24.7) | 1.06 |  |
| Other and unknown | 17.7 (14.6-21.3) | 0.77 |  |
| Simple transfusion |  |  |  |
| Rh (reference) | 3.9 (1.7-8.5) |  |  |
| ABO | 0.3 (0.2-0.6) | 0.08 |  |
| Other and unknown | 3.2 (1.9-5.3) | 0.80 | <0.0001 |
| Exchange fusion and/or IVIG |  |  |  |
| Rh (reference) | 1.0 (0.4-2.1) |  |  |
| ABO | 0.3 (0.1-0.5) | 0.26 |  |
| Other and unknown | 1.4 (0.5-3.6) | 1.46 | 0.04 |

CI, confidence interval; IVIG, intravenous immunoglobulin; OR, odds ratio.

**SUPPLEMENTAL FIGURE 1. HDFN alloimmunization type grouping**


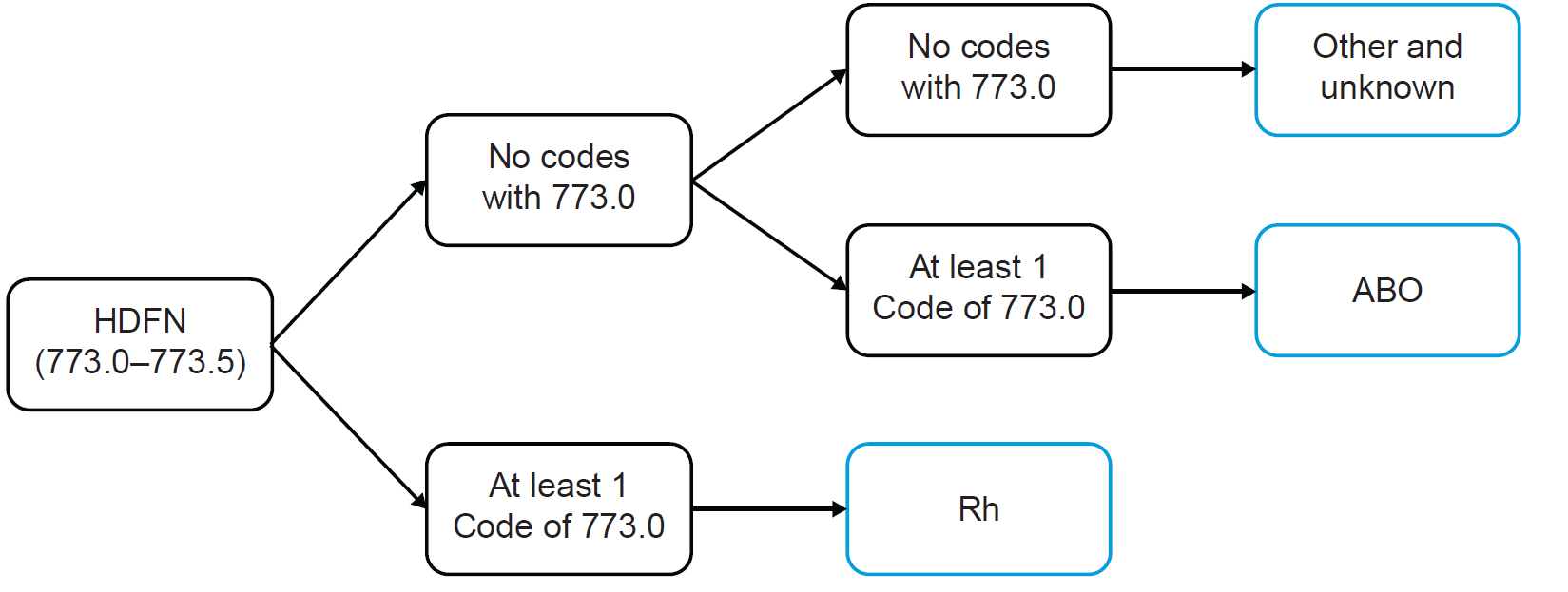


HDFN, hemolytic disease of the fetus and newborn.
